# Solid-State Fermentation with *Saccharomyces cerevisae*: A Strategy to Modulate the Properties of Faba Bean Flours

**DOI:** 10.3390/foods15173110

**Published:** 2026-09-01

**Authors:** Nerea Sarasa-Gil, Sandra Horvitz, María José Beriain, Paloma Vírseda, Francisco C. Ibañez, Débora Villaño

**Affiliations:** Institute on Innovation and Sustainable Development in Food Chain (IS-FOOD), Public University of Navarre (UPNA), 31006 Pamplona, Spain; nerea.sarasa@unavarra.es (N.S.-G.); mjberiain@unavarra.es (M.J.B.); virseda@unavarra.es (P.V.); pi@unavarra.es (F.C.I.); debora.villano@unavarra.es (D.V.)

**Keywords:** legumes, protein, sensory, *Vicia faba*, yeast

## Abstract

Faba bean (*Vicia faba* L.) is a high-protein legume limited by its sensory profile and antinutritional factors like RFOs (Raffinose Family Oligosaccharides). The impact of solid-state fermentation (SSF) with *Saccharomyces cerevisiae* on properties of starch-(SF) and protein-rich (PF) faba bean flours was investigated. Proximate composition, amino acid profile, digestible carbohydrates, organic acid contents, and techno-functional properties were determined. Sensory properties of SF- and PF-based crepes were also assessed. SSF reduced digestible carbohydrates by 10% (SF) and 22% (PF). RFOs concentration decreased by 39% in SF after SSF. Malic and acetic acids increased in both fractions. In PF, glutamic and aspartic acids increased (14.09 and 8.41%, respectively), whereas lysine decreased, suggesting protein structural modifications without compromising nutritional quality. Water absorption capacity rose by 12.41% (SF) and 6.7% (PF), while oil absorption capacity improved only in SF (22.63%). Water solubility decreased by 43.78% and 23.76% in SF and PF. Finally, compared to crepes made with unfermented flour, those prepared with fermented PF received a global acceptance 48.50% more favorable. These findings highlight SSF with *S. cerevisiae* as a promising strategy to improve sensory properties and expand the application of faba bean flour fractions as functional ingredients in plant-based food products.

## 1. Introduction

In recent years, the search for alternative protein sources has intensified in response to growing demand for plant-based diets and the need to reduce the environmental impact of food production [1,2]. Global plant-based food sales reached US$28.9 billion in 2025; maintaining 3% annual growth would yield approximately US$33.5 billion by 2030 [3]. Legumes represent one of the main sources of protein worldwide within the given context [4]. Furthermore, their tolerance to water and heat stress [5], combined with their capacity to reduce greenhouse gas emissions through biological nitrogen fixation [6], makes them particularly relevant for sustainable food systems.

Among legumes, soybean remains one of the primary crops utilized for global food and feed supply However, concerns related to its allergenicity, together with the regulatory requirements surrounding genetically modified organisms (GMO) in European Union and relatively low consumer acceptance of GMO-based foods, have encouraged interest in alternative grain legumes that can contribute to more sustainable European agricultural systems and reduce dependence on imported protein sources [7,8]. In this context, faba bean (*Vicia faba* L.) has emerged as a promising soybean alternative owing to its high nutritional value and adaptability to diverse agroecological conditions. Faba bean seeds contain approximately 27–34% protein and 40–47% starch on a dry basis, while total dietary fiber ranges from 15–27%. It is also a source of essential minerals, including potassium, phosphorus, magnesium, calcium, iron, and zinc, as well as vitamins, particularly folate. Its favorable protein content and amino acid profile further support its potential for use in plant-based food applications [9,10].

Protein-enriched legume flours are commonly obtained by air classification, a dry fractionation technique that separates the smaller protein-rich particles from the larger starch granules based on differences in size, shape and density [11]. This process yields two distinct fractions from the same grain, broadening the potential applications of faba bean in the food industry. While protein fractions are particularly valuable for use in meat analogues or nutritional supplementation, starch-enriched fractions can also improve the texture of different products, such as gluten-free alternatives [12]. However, the incorporation of faba bean ingredients into food formulations may be constrained by the presence of RFOs (Raffinose Family Oligosaccharides), which reduce digestibility [13], as well as by aroma and taste attributes commonly associated with unpleasant volatile compounds [6]. Thus, the development of faba bean-based products requires processing methods capable of improving their sensory, nutritional and techno-functional properties.

One such method is fermentation, a biotechnological process that involves the transformation of macromolecular components—carbohydrates, proteins, and lipids—into lower molecular weight products through the action of microbial enzymes [14,15]. In this regard, *Saccharomyces cerevisiae* has been reported to play a relevant role in sensory improvement due to the presence of enzymes such as alcohol dehydrogenase, which catalyze the conversion of aldehydes associated with beany off-flavors into less odor-active alcohols and esters, thereby enhancing the overall aroma and flavor of fermented substrates [16].

Fermentation can be carried out in submerged or solid-state conditions, differing in the medium in which the microorganisms grow [17]. By employing moist matrices with no free water, solid-state fermentation (SSF) requires less water and energy, has reduced operational costs, and its conditions closely resemble the natural microenvironment of many fermentative microorganisms [18,19]. The separate application of SSF to the protein and starch fractions of legume flours is of particular interest because their distinct compositions are expected to differentially influence microbial metabolism, and consequently the resulting nutritional profile and techno-functional properties.

Few studies have examined the effect of fermentation on legume flour fractions ob-tained by air classification [20,21,22], and to the best of our knowledge, no previous studies exist on the effect of SSF with *S. cerevisiae* on both fractions. This yeast is among the most widely used species in food fermentation and can contribute to improving legume characteristics through several mechanisms. In effect, *S. cerevisiae* has been reported to increase sulphur amino acid content in legume products [23,24]. Its primary metabolic activity involves carbohydrates, particularly monosaccharides, and produces organic acids and volatile compounds that can modulate flavor [25].

Therefore, the hypothesis of the study is that solid-state fermentation with *S. cerevisiae* is able to improve the properties of faba bean flours, thereby increasing their potential for use as ingredients in the production of plant-based products. Thus, two objectives have been proposed; firstly, to evaluate the impact of SSF with *S. cerevisiae* on nutritional and techno-functional properties of two faba bean flour fractions, one rich in protein and the second rich in starch. Secondly, to assess the impact of these changes on the sensory characteristics and acceptability of crepes made from these flours.

## 2. Materials and Methods

### 2.1. Materials

Starch- (SF) and protein- (PF) rich faba bean flours fractions were obtained by air classification process and were supplied by a local company (Sanygran SL, Tudela, Spain). Dry bakery yeast *Saccharomyces cerevisiae* (Lesaffre Panification, Maisons-Alfort, France) was used for the solid-state fermentation. α-Amylase and amyloglucosidase enzymes were purchased from Sigma-Aldrich (St. Louis, MO, USA). Commercial enzymatic assay kits for the determination of carbohydrates and organic acids were obtained from Biosystems (Barcelona, Spain), while the Raffinose/Sucrose/D-Glucose Assay Kit (K-RAFGL) was purchased from Megazyme (Wicklow, Ireland). All other reagents used in the analytical procedures were of analytical grade.

### 2.2. Solid-State Fermentation of Faba Bean Flours

**Figure 1:**
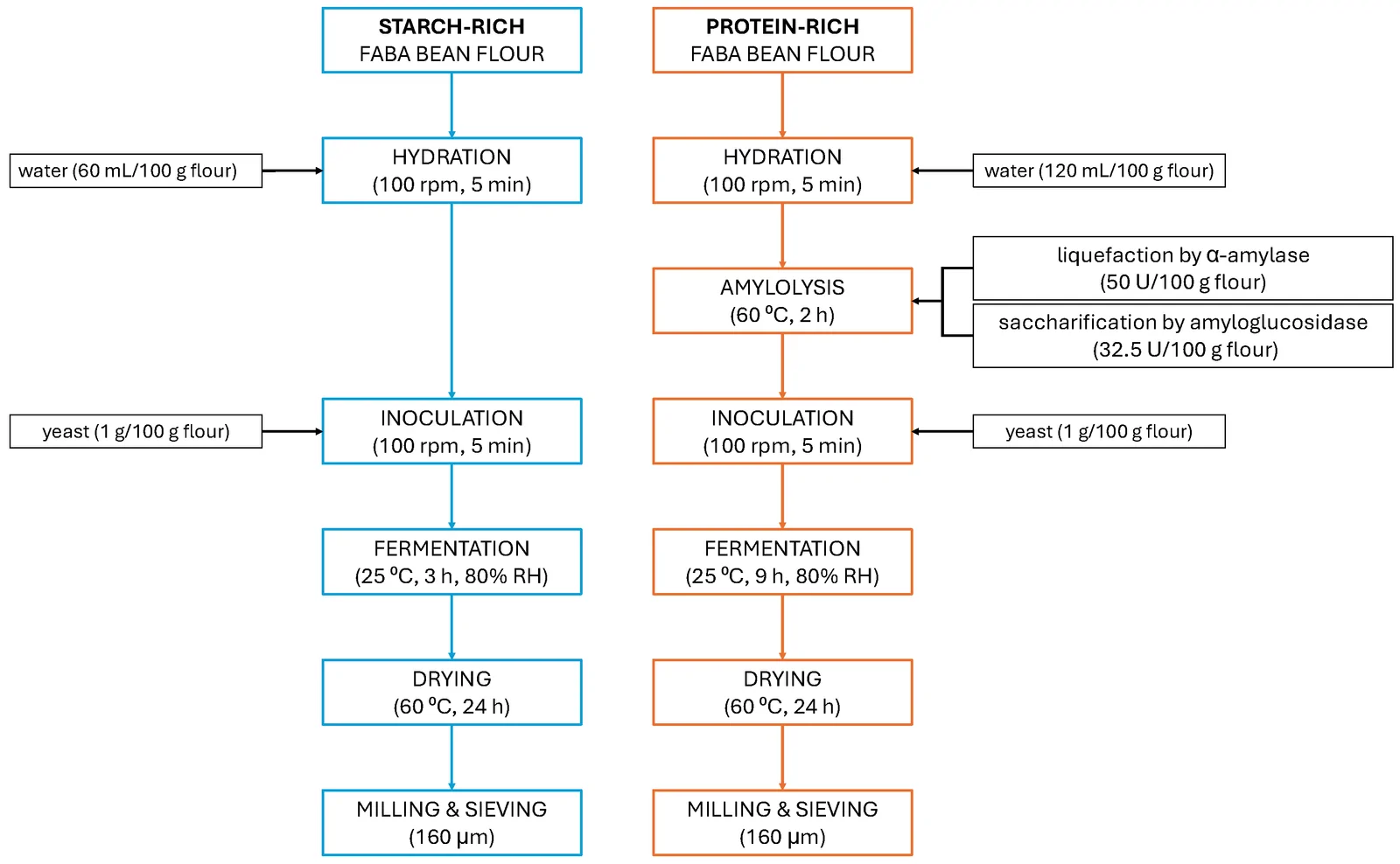
Treatments applied to starch-rich (**left**) and protein-rich (**right**) faba bean flours, including experimental conditions. Enzymatic hydrolysis (amylolysis) and subsequent enzyme inactivation were applied only to the protein-rich flour.

For activation, the yeast was diluted in water at 30 ± 0.2 °C (1 g/3 mL) and left to rest for 5 min before addition to the dough. The SSF process started by mixing the flour with water and adding the activated yeast. The dough was allowed to ferment and afterwards was dried, milled and sieved (Figure 1). An enzymatic hydrolysis (amylolysis) step was included for the protein-rich flour before mixing.

The SSF process was adapted for each fraction of faba bean fraction—SF or PF—as described below. The different water ratios were also selected according to the specific characteristics and processing requirements of each flour fraction.

#### 2.2.1. Treatment of SF

A homogeneous dough was obtained by mixing (5 min, 100 rpm) one kg of SF with water (600 mL) in a mixer (Spiral EVO, Bongard Iberia SA, Barcelona, Spain). Then the activated yeast was added (10 g/kg flour, diluted in 30 mL of warm water) keeping the mixing conditions for 5 additional min. The dough was distributed in flat aluminum trays (29 cm × 39 cm) to form a layer of approximately 1 cm thick and transported to a fermentation chamber with controlled temperature and humidity conditions (Iverpan 00 Salva Industrial SLU, Lezo, Spain). The fermentation process was carried out for 3 h at 25 ± 1 °C and 80 ± 2% humidity [26]. Afterwards, the SF was dried at 60 ± 1.5 °C for 20 h in a drying chamber (J. P. Selecta, Barcelona, Spain). The dry product was milled and sieved using a laboratory mill (CD1 Mill, CHOPIN Technologies, Villeneuve-la-Garenne, France) to obtain flour with a maximum particle size of 160 μm. The flour was packaged in vacuum-sealed bags until analysis.

#### 2.2.2. Treatment of PF

Prior to fermentation and to increase the availability of fermentable carbohydrates for *S. cerevisiae*, the starch present in the PF was hydrolyzed using α-amylase and amyloglucosidase enzymes from Megazyme kit, following the method of Ait Chekdid et al. [27]. One kg of PF was mixed with water (1.2 L) using a mixer (Spiral EVO, Bongard Iberia SA, Barcelona, Spain) at 100 rpm until full homogenization. The dough was then transferred to a Thermomix (Vorwerk TM5, Wuppertal, Germany) and heated to 60 ± 1 °C under continuous stirring. α-amylase (50 U/100 g flour) was added first and left to act for 15 min, followed by amyloglucosidase (32.5 U/100 g flour) for 105 min, both at 60 °C under continuous stirring. The dough was then cooled to 30 ± 1 °C, and the activated yeast (10 g/kg flour, diluted in 30 mL of warm water) was added and left to ferment at 25 ± 1 °C and 80 ± 2% relative humidity in a fermentation chamber (Iverpan 00, Salva Industrial SLU, Lezo, Spain).

During SSF, samples were taken every 60 min and the total reducing sugar concentration was measured using the DNS method [28]. Fermentation was considered complete after 9 h, when the rate of sugar consumption decreased, suggesting a reduced metabolic activity. After fermentation, the added enzymes were inactivated by heating at 90 ± 1 °C for 10 min in an oven (SCC 61, Rational AG, Landsberg am Lech, Germany), and the product was dried, milled and sieved following the same procedure as for the SF flour.

Figure S1 shows the appearance of the flours utilized in this study.

### 2.3. Characterization of Faba Bean Flours

Faba bean flours were characterized before and after SSF. The four flour types were coded as follows: unfermented protein-rich faba bean flour (UPF), fermented protein-rich faba bean flour (FPF), unfermented starch-rich faba bean flour (USF) and fermented starch-rich faba bean flour (FSF).

#### 2.3.1. Physicochemical Properties

Prior to chemical analysis pH was determined. Each flour sample was dispersed (1 g) in 10 mL of deionized water and pH was measured by a pH-meter (BASIC 20, Crison Instruments SA, Barcelona, Spain) equipped with a pH probe (pH5202, Crison Instruments SA, Barcelona, Spain).

The proximate composition of the four flours was determined using the Association of Official Analytical Chemists (AOAC) standard methods 925.10 (moisture), 923.03 (ash), 920.87 (total nitrogen content) and 922.06 (raw fat) [29](AOAC, 2005). A factor of 5.7, specific for legumes, was employed for estimation of raw protein based on N content in the flours [30]. The carbohydrate content was calculated by difference. Data were expressed as g of each compound per 100 g of flour on a dry basis.

#### 2.3.2. Determination of Digestible Carbohydrate and Organic Acid Contents

The concentrations of total starch, sugars (maltose, sucrose, lactose, glucose, fructose, galactose), and organic acids (L-lactic, D-lactic, malic, citric, and acetic acids) were quantified using commercial enzymatic assay kits (Biosystems, Barcelona, Spain) in an automatic analyzer (A15 Biochemistry Analyzer, Biosystems, Barcelona, Spain). Data were expressed as g of each compound per 100 g of flour dry weight.

Prior to quantification, the analytes were extracted following the protocols provided by the kit manufacturers. For each sample, 5 g of flour were weighed and suspended in 25 mL of ultrapure MilliQ water (Poseidon Ultrapure, Pamplona, Spain). The suspension was subjected to ultrasound treatment (40 kHz frequency, 180 W power) for 5 min to enhance extraction, followed by incubation at 50 °C for 15 min. Samples were then centrifuged at 10,500× *g* for 10 min (Jouan MR 18.22, Labexchange, Burladingen, Germany) and the supernatant was collected. For protein precipitation, 5 mL of Carrez I reagent were added and mixed thoroughly, followed by 5 mL of Carrez II reagent with additional mixing. The pH was adjusted to 7.5 using 1 N NaOH, and the volume was brought up to 100 mL with ultrapure MilliQ water. A second centrifugation step was carried out at 2500× *g* for 10 min, and the resulting supernatant was used for analysis.

#### 2.3.3. Determination of Raffinose Family Oligosaccharides Contents

Total RFOs (Raffinose Family Oligosaccharides) were determined using the Raffinose/Sucrose/D-Glucose Assay Kit from Megazyme. Flour samples (0.5 g) were extracted with 95% ethanol, incubated at 86 ± 2 °C for 5 min to inactivate endogenous enzymes, diluted with 50 mM sodium acetate buffer (pH 4.5) and extracted for 15 min under continuous mixing (LanTechnics, Shangai, China). This solution (5 mL) was treated with chloroform (2 mL) and centrifugated at 1000× *g* for 10 min. Aliquots (0.2 mL) of the aqueous phase were incubated separately with sodium acetate buffer, invertase, or α-galactosidase plus invertase to determine glucose, glucose + sucrose, and glucose + sucrose + RFOs, respectively. After incubation at 50 °C for 20 min, GOPOD reagent was added and absorbance was measured at 510 nm (spectrophotometer, Thermo Fisher Scientific Inc., Madrid, Spain). RFOs content was calculated by difference and expressed as g/100 g flour on dry basis using the molecular weight of verbascose (828.7 g/mol) for conversion from mmol to grams, as this compound has been reported as the predominant RFOs in faba bean [31].

#### 2.3.4. Amino Acid Profile

The amino acid composition of the flours was determined by Eurofins Nordeste SL (Pamplona, Spain) following the official method ISO 13903:2005 [32]. Briefly, acid hydrolysis was performed with 6 M HCl at 110 °C for 24 h. For the quantification of sulphur-containing amino acids—cysteine and methionine—samples were oxidized at 0 °C with a performic acid/phenol mixture prior to hydrolysis; excess reagent was decomposed with sodium bisulphite, and samples were subsequently hydrolyzed with 6 M hydrochloric acid for 23 h. Prior to analysis, the pH was adjusted to 2.2 and samples were filtered through a 0.22 µm membrane filter (Millipore, Merck KGaA, Darmstadt, Germany). Amino acids were separated by ion-exchange chromatography (IC-UV) and detected by post-column reaction with ninhydrin using photometric detection at 570 nm, except for proline, that was determined at 440 nm. Tryptophan content was assessed according to the official method EU 152/2009 [33], based on alkaline hydrolysis followed by liquid chromatography with fluorescence detection (LC-FLD). Amino acid concentrations were expressed as g of each amino acid per 100 g of flour on a dry weight basis.

### 2.4. Techno-Functional Properties of Faba Bean Flours

#### 2.4.1. Water Properties

Water absorption capacity (WAC) and water solubility (WS) were determined following the methods described by Dundar and Gocmen [34]. Each flour sample (0.5 g) was dispersed in 5 mL of distilled water and vortexed (Movil-Vib, JP Selecta S.A., Barcelona, Spain) for 15 s every 5 min, over a 40 min period. The suspension was then centrifuged at 2100× *g* for 10 min (Sigma 3K30, Sigma Laborzentrifugen, Osterode am Harz, Germany). The supernatant was decanted into a pre-weighed evaporating dish, and the sediment was weighed to estimate its solids content. Dry solids from the supernatant were recovered by evaporation overnight at 105 °C. WAC and WS were calculated as the ratio of absorbed water to sample weight (WAC) and the ratio of dissolved solids to total solids (WS), expressed as percentages (Equations (1) and (2), respectively):

(1) $$\mathrm{WAC}\left(\%\right)=\;\frac{\mathrm{weight}\;\mathrm{of}\;\mathrm{wet}\;\mathrm{precipitate}\left(g\right)\;-\;\mathrm{weight}\;\mathrm{of}\;\mathrm{dried}\;\mathrm{precipitate}\left(g\right)}{\mathrm{weight}\;\mathrm{of}\;\mathrm{sample}\left(g\right)},$$

(2) $$\mathrm{WS}\left(\%\right)=\frac{\mathrm{weight}\;\mathrm{of}\;\mathrm{dried}\;\mathrm{supernatant}\left(g\right)}{\mathrm{weight}\;\mathrm{of}\;\mathrm{wet}\;\mathrm{precipitate}\left(g\right)}\times 100.$$

#### 2.4.2. Oil Property

Oil absorption capacity (OAC) was determined following the method of Zhu et al. [35]. One gram of each flour was added to 10 mL of sunflower oil and vortexed for 30 s. The suspension was left to stand for 30 min at room temperature (20 ± 2 °C) and then centrifuged at 1100× *g* for 20 min. The supernatant was collected and weighed. OAC was calculated as the difference between the weight of oil added and the weight of recovered supernatant, relative to sample weight, expressed as a percentage (Equation (3)):

(3) $$\mathrm{OAC}\left(\%\right)=\;\frac{\mathrm{weight}\;\mathrm{of}\;\mathrm{added}\;\mathrm{oil}\left(g\right)\;-\;\mathrm{weight}\;\mathrm{of}\;\mathrm{supernatant}\left(g\right)}{\mathrm{weight}\;\mathrm{of}\;\mathrm{sample}\left(g\right)}\;\times \;100.$$

#### 2.4.3. Emulsifying Properties

Emulsifying properties were assessed according to Yasumatsu et al. [36]. Each flour sample (4.2 g) was mixed with 60 mL of distilled water and 60 mL of sunflower oil using an Ultra Turrax T25 homogenizer (IKA, Staufen, Germany). The mixture was homogenized by progressively increasing the speed to 10,000 rpm, which was maintained for 90 s. The emulsion was equally distributed into three 50 mL centrifuge tubes and centrifuged at 1300× *g* for 5 min (Sigma 3K30, Sigma Laborzentrifugen, Osterode am Harz, Germany). Emulsifying activity (EA) was calculated as the ratio of the emulsified layer volume (V_E_) to the total volume of the mixture prior to centrifugation (V_T_), expressed as a percentage (Equation (4)). When an emulsion was formed, emulsion stability (ES) was assessed using the same samples. The emulsions were heated at 80 °C for 30 min, cooled to room temperature (20 ± 2 °C), and centrifuged at 1300× *g* for 5 min. The volume of the remaining emulsified layer (V_E80_) was recorded. ES was expressed as the percentage of the emulsion layer volume remaining after heating (V_E80_) relative to the total initial volume (V_T_) (Equation (5)):

(4) $$\mathrm{EA}\left(\%\right)=\frac{V_{E}\;\left(\mathrm{mL}\right)}{V_{T}\;\left(\mathrm{mL}\right)}\times 100,$$

(5) $$\mathrm{ES}\left(\%\right)=\frac{V_{E80}\;\left(\mathrm{mL}\right)}{V_{T}\;\left(\mathrm{mL}\right)}\times 100.$$

#### 2.4.4. Foaming Properties

Foaming capacity (FC) and foam stability (FS) were determined following the method described by Silva et al. [37]. Each flour (2.5 g) was dispersed in distilled water to a final concentration of 2.5% (*w*/*w*). Aliquots of 15 mL (V_0_) were homogenized using an Ultra Turrax T25 (IKA, Staufen, Germany) at 15,000 rpm for 2 min. FC was expressed as the percentage increase in volume after homogenization relative to the initial dispersion volume (V_0_). FS was expressed as the percentage of foam volume remaining after standing for 30 min at room temperature (V_2_) relative to the total volume immediately after homogenization (V_1_) (Equations (6) and (7)):

(6) $$\mathrm{FC}\left(\%\right)=\frac{V_{1}\;\left(\mathrm{mL}\right)\;-{\;V}_{0}\;\left(\mathrm{mL}\right)}{V_{0}\;\left(\mathrm{mL}\right)}\times 100,$$

(7) $$\mathrm{FS}\left(\%\right)=\frac{V_{2}\;\left(\mathrm{mL}\right)}{V_{1}\;\left(\mathrm{mL}\right)}\times 100.$$

#### 2.4.5. Gelation Property

The least gelation concentration (LGC) was determined following the method described by Silva et al. [37]. Flour dispersions were prepared at concentrations of 2, 4, 6, 8, 10, 12, 14, 16, 18, and 20% (*w*/*v*) in a final volume of 5 mL. The dispersions were heated at 95 °C for 60 min, rapidly cooled on ice for 5 min, and stored overnight at 4 °C. The following day, tubes were inverted and gel consistency was classified as: (1) liquid—no gel formation; (2) weak gel—the dispersion flowed with some resistance; (3) gel—the dispersion did not flow upon inversion. LGC was defined as the minimum flour concentration required to form a stable gel.

### 2.5. Elaboration of Crepes

Five crepe formulations were prepared for the sensory evaluation of the faba bean flours. The control formulation consisted of 125 g of wheat flour, 1 egg, 10 g of sugar, and 250 mL of whole milk. In the experimental formulations, wheat flour was partially (30%) or fully (100%) replaced with fermented or unfermented protein-rich and starch-rich faba bean flour fractions, respectively. Detailed ingredient compositions are presented in Table 1. A total of six crepes were produced for each formulation. Crepes were cooked in a lightly oiled frying pan for 2 min per side.

### 2.6. Sensory Evaluation of Crepes

The sensory evaluation was conducted in the sensory laboratory facilities of the Public University of Navarra (UPNA). Laboratory staff members with experience in food sensory analysis were recruited as judges (n = 16; age range: 25–56 years). All the samples were coded with random three-digit numbers following a Latin square design to ensure blinding, and one sample of each crepe type (wheat, USF, FSF, UPF, FPF) was presented to each panelist. Evaluation sessions were conducted under white light, in temperature-, and humidity-controlled booths. The study was approved by the UPNA Ethics Committee (Code: PI-027/25). Figure S2 shows the appearance of three examples of crepes that were used in the sensory assessment.

Sensory attributes were assessed using a five-point hedonic Likert scale, including appearance, aroma, taste, consistency, and stickiness. Global acceptance was rated on a nine-point, numerical hedonic scale, where 1 represented the lowest and 9 the highest score [38]. Appearance was defined as the absence of cracks and surface roughness, with a characteristic uniform color. Attributes for odor were evaluated by odor intensity and the presence of off-odors. Taste was assessed as pleasant, free from strange aftertaste, and characteristic of legumes. Consistency was described as uniform, smooth, firm, and chewable. Stickiness was also included as a relevant textural parameter for this type of product.

### 2.7. Statistical Analysis

All analytical measurements were performed in triplicate and were reported as the mean ± standard deviation. Nonparametric methods were employed due to the small sample size of all analyses (n < 30) and the discrete data from the sensory analysis. The effect of SSF on the physicochemical and techno-functional properties was evaluated using a bilateral Mann-Whitney U test for unpaired samples (α = 0.05), with each flour fraction (SF and PF) analyzed independently. For the sensory analysis, wheat was included as a control in the statistical analysis. Kruskal-Wallis’s H test (α = 0.05) was performed separately for the wheat control, unfermented SF, and fermented SF, and for the wheat control, unfermented PF, and fermented PF. When the Kruskal-Wallis’s test was significant, the mean pairs were compared using the Mann-Whitney test (α = 0.05). Data were subjected to statistical analysis using IBM SPSS Statistics (Version 30, IBM Corporation, New York, NY, USA).

## 3. Results

### 3.1. Changes in Physicochemical Properties of Flours

The proximate composition of the unfermented and fermented starch- and protein-rich faba bean flour fractions is reported in Table 2. Both fractions exhibited similar trends in proximate composition following fermentation. Fat contents remained unchanged in SF and slightly increased in PF, whereas carbohydrate content decreased significantly in both fractions after fermentation (3.61% and 14.88% in SF and PF, respectively). Total protein and ash content increased significantly in SF flour but decreased in PF flour.

### 3.2. Changes in Digestible Carbohydrates and Organic Acids Content of Flours

The digestible carbohydrates (starch, mono, and disaccharides) and organic acids content in the faba flours, before and after the fermentation process is shown in Table 3. After fermentation, the total digestible carbohydrates content decreased by 10% and 22% in the starch-rich and the protein-rich flours, respectively. Sucrose significantly decreased in both fractions while maltose and starch concentrations were significantly reduced only in the protein-rich fraction. After fermentation, lactose only increased slightly in the starch-rich fraction (from 0.00 to 0.02 g/100 flour DW) while it was undetectable in the protein-rich fraction, either before or after the fermentation process. In parallel, the monosaccharides glucose, fructose and galactose increased in both flours after fermentation, reaching an increase of up to 5-fold in the starch-rich flour.

The fermentation process significantly reduced the raffinose oligosaccharides in the starch-rich fraction by 39% and a numerical decrease in RFOs was also observed in PF; however, this reduction was not statistically significant.

Regarding organic acids, after fermentation of the starch-rich fraction, a significant increase in malic, L-lactic and acetic acid was observed, although at minor levels, together with a decrease in citric acid concentration. In this flour, D-lactic acid was not detected either in the unfermented or in the fermented samples. On the other hand, in the fermented protein-rich flour, only the malic acid increased significantly while the D-lactic acid decreased, and no significant changes were detected for the citric, L-lactic and acetic acids. Although these observed changes were statistically significant, the resulting quantities were practically negligible.

### 3.3. Changes in Amino Acid Profile of Flours

The amino acid composition of both unfermented and fermented starch- and protein-rich faba bean flour fractions is presented in Table 4. Within each fraction, statistical comparisons were performed between unfermented and fermented samples. Subsequent to the fermentation of starch-rich flours, an increase in the concentration of five amino acids was observed. Conversely, in protein-rich flours, only three amino acids exhibited an increase. This increase was significant for aspartic acid and glutamic acid in both fractions. Lysine increased in starch-rich flour but decreased in protein-rich flour. A significant change in the content of sulphur-containing amino acids was also observed, but only in the starch-rich flour, with an increase in methionine levels, parallel to a reduction in the levels of cysteine and its dimer cystine. The present study revealed a statistically significant rise in total amino acid content, with an 18.9% increase in FSF samples and a 9.9% increase in FPF samples.

### 3.4. Changes in Techno-Functional Properties of Flours

The functional properties of the flours before and after fermentation are summarized in Table 5.

Regarding hydration properties, fermentation significantly increased Water Absorption Capacity (WAC) in both SF (12.41%) and PF (6.71%), while Water Solubility (WS) decreased significantly in both fractions (43.78 and from 23.76% in SF and PF, respectively). Oil Absorption Capacity (OAC) increased significantly after fermentation in SF, whereas it remained unchanged in the protein-rich fraction.

About emulsifying properties, fermentation decreased both Emulsifying Activity (EA) and Emulsifying Stability (ES) in SF, while it only decreased ES in PF. Foaming properties were differently affected depending on the fraction, as Foaming Capacity (FC) decreased in both fractions but only significantly in PF (18.92%), whereas Foaming Stability increased also in both, but only significantly in SF (13.89%) after fermentation.

Table 6 shows the least gelling concentration of both flour fractions before and after fermentation, assessed at concentrations ranging from 2 to 20% (*w*/*v*) in water. SF showed limited gelling capacity, with only FSF forming a gel at a 20% concentration, while PF exhibited greater gelling ability (LGC = 12%), which was not affected by fermentation.

### 3.5. Sensory Evaluation of Crepes

**Figure 2:**
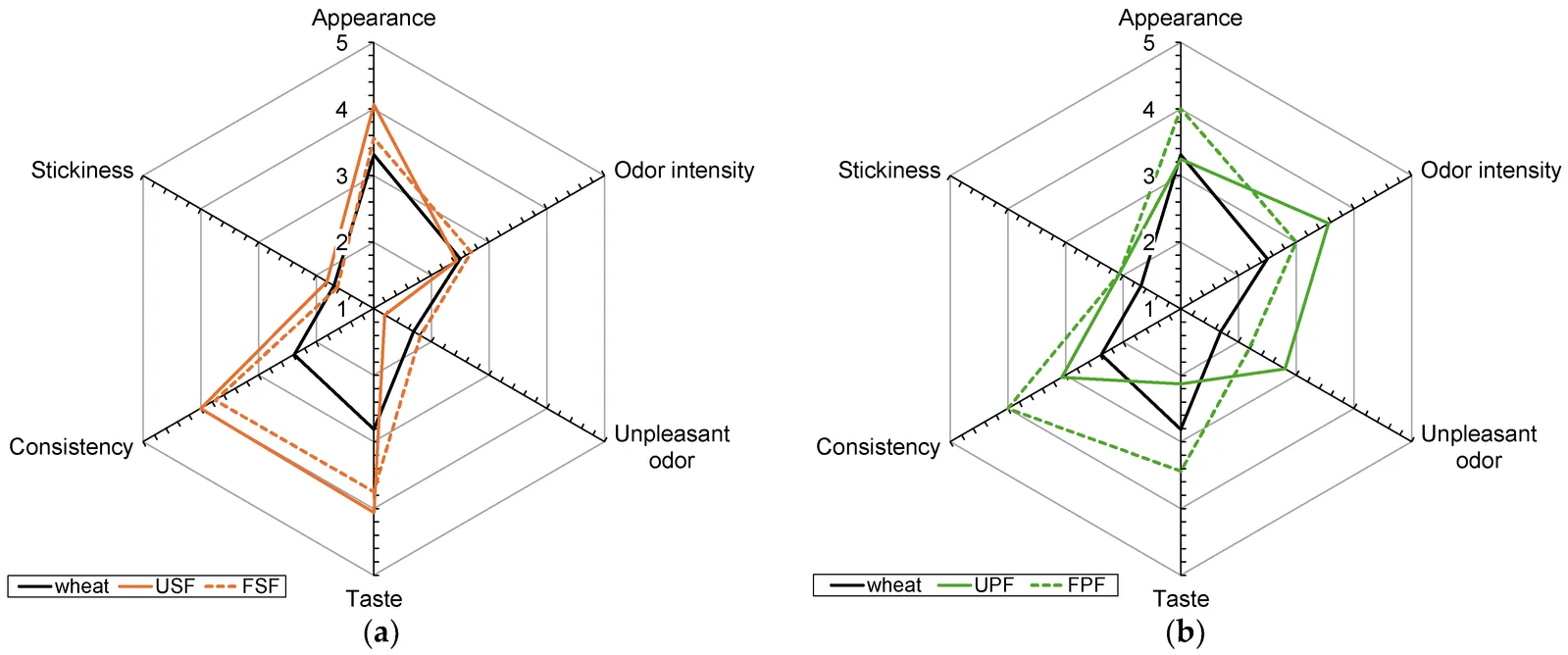
Sensory quality attributes of crepes formulated with wheat flour (control) and faba bean flour fractions evaluated using a structured scale, including appearance, odor intensity, unpleasant odor, taste, consistency, and stickiness. (**a**) wheat flour compared with USF and FSF; (**b**) wheat flour compared with UPF and FPF. USF: unfermented starch flour; FSF: fermented starch flour; UPF: unfermented protein flour; FPF: fermented protein flour.

Crepes were prepared by replacing wheat flour totally (100%) with SF or partially (30.0%) with PF. Crepes prepared with 100% wheat flour were used as controls. The sensory attributes evaluated are shown in Figure 2 and Tables S1 and S2.

In crepes made with SF fraction, fermentation only affected unpleasant odor, increasing it. When compared to the control wheat flour, neither USF nor FSF differed significantly in odor intensity, unpleasant odor, or stickiness; however, both received significantly higher scores for taste, consistency and global acceptance; while USF also exhibited a more favorable appearance. In contrast, fermentation had a marked effect on several sensory attributes in crepes made with PF fractions. Compared with the control wheat flour, UPF-based crepes showed significantly higher odor intensity and unpleasant odor, and significantly lower taste and consistency scores. These attributes improved after fermentation: odor intensity decreased significantly in FPF relative to UPF, while taste and consistency increased significantly; however, all three attributes remained significantly different from the control in FPF crepes.

Regarding global acceptance, both USF and FSF crepes scored significantly higher than the control (6.75 ± 1.34 and 6.31 ± 1.25, respectively vs. 4.63 ± 1.36), but fermentation did not produce significant changes in crepes made with starch-rich fraction. On the other hand, in PF-based crepes, global acceptance did not differ significantly from the control in UPF (4.00 ± 1.10 vs. 4.63 ± 1.36), but increased significantly following fermentation, to 5.94 ± 1.53 in FPF.

## 4. Discussion

In this study, a starch-rich and a protein-rich faba bean flour fraction was fermented with *S. cerevisiae* and the effects on the nutritional composition, techno-functional properties and sensory attributes were investigated. Unfermented protein-rich flour showed a 50% content in protein, two fold higher compared with the typical protein content of whole faba bean flours (25–30%) [39,40,41]. In this fraction also the fat and ash contents were higher than in the whole flours, what is consistent with the results reported by Xing et al. [12], who argue that starch granules are easily separated into the starch-rich fraction, pushing other components (like protein, oil and ash) into the protein fraction. On the other hand, the carbohydrate content in the starch-rich fraction was higher than in the whole faba bean flour (60–68%) [40,42] and almost doubled the carbohydrates content in the protein-rich fraction. This confirms the significant compositional differences due to air fractionation process, between the two types of faba bean flour used in the study.

Following fermentation, significant changes observed for raw protein and raw fat content were less than one unit of measurement and the impact on these variables can be considered irrelevant. Raw carbohydrate content decreased significantly in both fractions, with a more pronounced reduction in PF. Regarding ash content, it increased after fermentation in SF, in agreement with the results reported by Kasprowicz-Potocka et al. [23], whereas it decreased in the PF, which could be attributed to the formation of chelates with proteins or with the citric acid present in the flour [43].

*S. cerevisiae* cannot degrade starch, the main carbohydrate found in SF, as it lacks specific hydrolytic enzymes. Instead, this yeast utilizes sucrose as an energy substrate, which is extracellularly degraded by an invertase into glucose and fructose [44]. Maltose can also be hydrolyzed by α-glucosidase (maltase) in the yeast cell, rendering two molecules of glucose. Consequently, in SF, no significant changes were observed in starch concentration, while there was a significant decrease in sucrose concentration, and a slight, non-significant reduction in maltose. Conversely, PF underwent a pretreatment with α-amylase and amyloglucosidase enzymes prior to fermentation, which contributed to degrading starch into maltose and glucose, which were further metabolized by the yeast [45]. Glucose is the preferred substrate for *S. cerevisiae*, over other monosaccharides or carbon sources [46]. The glucose molecules of available sugars were greater in PF than in SF and the yeast predominantly utilized them, leading to a significant reduction in their concentration. The decrease observed in disaccharides such as maltose and sucrose in both fractions, have also been reported in other studies [47].

Regarding changes in RFOs, the rate of their utilization during fermentation depends on initial substrate conditions: in substrates with high glucose content, RFOs utilization is comparatively lower [48]. This would explain the limited RFOs degradation observed in PF, where the high availability of fermentable sugars likely reduced yeast use of RFOs as an energy source. In contrast, in the SF fraction no hydrolytic enzymes were added prior to fermentation, and the initial starch content was high, resulting in lower initial glucose levels. Under these conditions, yeast growth relied more heavily on sucrose and RFOs as fermentable substrates, leading to a reduction in their concentrations and a parallel increase in monosaccharide levels. The observed rise in galactose concentration is consistent with the hydrolysis of RFOs such as raffinose and stachyose, antinutritional factors commonly present in legumes. Kasprowicz-Potocka et al. [23] reported that raffinose oligosaccharides were completely consumed during yeast fermentation of lupin seeds, whilst Tudor et al. [49] and Amadei et al. [47] [] also observed a significant reduction of these compounds when fermenting soybean and chickpea flours with *S. cerevisiae*, respectively. Yeast degrades these oligosaccharides into simple sugars by the production of enzymes as α-D-galactosidase [50]. *S. cerevisiae* possesses *MEL* genes encoding α-galactosidases and can cleavage the α-1,6 bonds in RFOs structure [51,52]. In parallel, yeast metabolism increases the concentration of organic acids, lowering the pH. This acidification of the medium may activate endogenous α-galactosidases naturally present in the legume, thereby contributing to the decrease in RFO levels [53]. The reduction observed could improve the digestibility of faba bean flours, since these oligosaccharides are not hydrolysable by human enzymes and are consequently fermented by the gut microbiota, causing flatulence and gastrointestinal discomfort [12].

Concerning organic acids, minor levels of malic and acetic acid were detected; only citric acid showed substantial levels that decreased with fermentation, what can be related to its use as energy source by the yeast, in accordance with the results reported by Munch-Andersen et al. [54].

Increases in all amino acids were observed after fermentation in both flour fractions, that were significant for aspartic and glutamic acids, as well as lysine. Some authors have reported increases in certain amino acids following fermentation of blue lupin seeds with *S. cerevisiae*, attributed to an increase in biomass production [23]. Yeast biomass may be preferentially enriched with certain amino acids, especially those derived from TCA cycle, as glutamate [55]. The apparent increase in aspartic and glutamic acid concentrations can be attributed to a sequential process initiated by medium acidification. The accumulation of organic acids alters the net charge of the proteins, disrupting stabilizing electrostatic interactions and inducing conformational modifications that lead to partial protein unfolding. This reduction in pH shifts the environment toward the optimum catalytic range of endogenous plant proteases triggering their activation. These active proteases cleave the complex, unfolded protein matrix into accessible smaller peptides. Finally, the synergistic effect of the acidic environment and the increased molecular exposure accelerate a non-enzymatic deamidation of the amide side chains in the exposed asparagine and glutamine residues, converting them chemically into aspartic and glutamic acids, respectively [56,57]. On the other hand, it has been described that *S. cerevisiae* can assimilate inorganic N, as ammonium ions, and convert them into glutamate and glutamine, which act as universal nitrogen donors for the synthesis of all other amino acids. Besides, using glucose as an energy source in glycolysis, yeast generates carbon skeletons to produce essential amino acids that can be scarce initially, as lysine or branched-amino acids (Leu, Ile, and Val) [58]. In addition, there was an increase of methionine in the FSF. The methionine deficiency that occurred in SF stimulates the fungal synthesis of this essential amino acid, resulting in a significant increase after fermentation, whereas PF does contain this amino acid and no significant changes were observed. Methionine synthesis in fungal, bacteria and plants is carried out by different sulfur metabolic pathways. It can be synthesized from homoserine by direct sulfhydrylation, as well as from other sulphur amino acids via trans-sulphuration pathway [59]. In the present study, it is possible that cysteine and its dimer cystine, were converted into methionine by this last mechanism of trans-sulphuration, but this hypothesis needs to be confirmed in specific studies.

Specific reduction on lysine on PF fraction may be due to a differential proteolysis followed by preferential lysine metabolization, rather than proteolysis as the direct cause of lysine destruction. The balance is contingent upon the fungal species, substrate, and experimental conditions for fermentation [60].

Techno-functional properties were evaluated in both flour fractions prior and after fermentation. The observed decrease in Water Solubility (WS) and the increase in Water Absorption Capacity (WAC) in both flours is consistent with previous studies on fermented legume-based matrices, such as pea protein-enriched flour and chickpea flour [61,62]. In the FSF, the increase in WAC can be explained by two closely related mechanisms: modification of starch-granule architecture and greater exposure of hydrophilic groups. Although *S. cerevisiae* cannot hydrolyze starch directly, the acidic environment generated during fermentation, together with the metabolic activity of the flour’s endogenous amylases, may progressively modify starch-granule structure, particularly the amorphous region, making it more accessible for water to penetrate the granules. Conversely, the damage to the starch led to an increase in the accessibility of the molecule’s hydroxyl groups, thereby enhancing its capacity for water absorption [63,64]. Regarding WS, the larger relative decrease in SF (43.8%) also suggests an important contribution of changes in starch structure, including disruption of its semi-crystalline organization and possible depolymerization during fermentation. In the FPF, other factors must also be taken into account. The heat treatment at 90 °C applied after fermentation may have caused partial denaturation of the proteins, unfolding of the globulin structures and exposure of hydrophilic groups [65]. This phenomenon would explain the increase in WAC; furthermore, it may have led to the formation of aggregates, which in turn could explain the decrease in WS [66,67,68]. Also, the decrease in pH in both fractions after fermentation (to approximately 5.8), may have promoted protein–protein interactions and aggregation by modifying protein charge.

Changes in Oil Absorption Capacity (OAC) could also be related to starch hydrolysis. Structural disruption, including the loss of crystallinity, make starch granules more porous and less compact, facilitating the absorption of both oil and water [15,62,69]. In SF, this increase in OAC may be explained by a larger exposed starch surface area, more irregular particle morphology, and greater exposure of lipophilic glucan chains, all of which enhance oil retention through capillary networks and surface oleophilicity. Residual lipids may also interact with amylose to form starch–lipid complexes, although highly stable complexes could instead limit oil uptake by increasing matrix density [70]. OAC increase could be also related to the increase in hydrophobic amino acids, especially in SF. Proteins containing a greater proportion of hydrophobic amino acids, such as leucine, isoleucine, valine, and phenylalanine, all of which increased after fermentation, can interact with nonpolar lipids via van der Waals forces [65]. This effect would enhance protein–lipid interactions by facilitating direct binding with the hydrocarbon chains of fatty acids in oil [62,69]. Furthermore, the aggregation of unfolded proteins into clusters could create microcapillary spaces that enable the physical entrapment of oil [15].

For industrial applications, increased OAC and WAC are desirable in plant-based formulations, as they contribute to reduce water separation, lower cooking losses, and improve texture and mouthfeel [71]. However, scaling up this solid-state fermentation process would require careful control of heat and mass transfer, as heat generation, O_2_/CO_2_ exchange, and moisture loss become increasingly difficult to manage at larger bed volumes, which could compromise the reproducibility of the techno-functional profile obtained at laboratory scale [72,73].

Regarding emulsion properties, in PF both Emulsion activity (EA) and stability (ES) tended to increase after fermentation. The heat treatment at 90 °C may have induced partial denaturation of globulin structures, exposing hydrophobic regions that enhance interfacial adsorption at the oil-water interface [65]. In SF these properties decreased significantly, which could be associated with the marked reduction in solubility observed in this fraction, as well as with the lower starch content, since starch can contribute to emulsion stabilization by increasing viscosity [74].

Foaming properties were also influenced by fermentation. The decrease in foaming capacity (FC) may be associated with the reduced solubility observed in the fermented flours, given the strong dependence of foaming behavior on protein solubility [15]. On the other hand, Foaming Stability (FS) increased in both fractions, with a significant improvement observed in the starch-rich fraction. This enhancement in FS may be partly attributed to the free sugars generated during fermentation, which can increase the viscosity of the continuous phase and thereby slow drainage and bubble coalescence, stabilizing the foam structures [75].

Finally, gelation properties reflected differences in protein content between fractions. Gelation takes place when proteins assemble into a three-dimensional network that resists deformation under pressure [11]. Therefore, the limited gelation capacity observed in SF can be explained by their lower protein content. PF maintained its gelling capacity after fermentation, with a Least Gelling Concentration (LGC) of 12%. Contradictory results have been reported in the literature regarding the effect of fermentation on gelling properties. While fermentation of quinoa flour with *Aspergillus oryzae* and *Rhizopus oligosporus* did not alter gelling capacity [76], fermentation of faba bean flour reduced the minimum gelation concentration [77], suggesting that the impact of fermentation on these properties is highly dependent on the substrate composition and the fermenting microorganism used [78].

The sensory study revealed that fermentation had a positive impact on the organoleptic characteristics of the crepes elaborated with protein-rich faba bean flour fraction. The improved acceptability of crepes made with the fermented protein-rich fraction (FPF), compared with those made with the unfermented fraction (UPF), may be attributed to their enhanced taste and consistency, as well as their lower odor intensity, as reported by the panelists. These sensory improvements may be related to the release of sugars, which could contribute to sweetness and help mask unpleasant aromas. In particular, the enzymatic pre-treatment and fermentation generated substantial amounts of free glucose and fructose. These monosaccharides participate in Maillard reactions and caramelization during cooking, generating pleasant roasted and caramel-like volatile compounds that may further mask residual beany off-notes. Fermentation had a more pronounced impact on the sensory properties of protein-rich fraction, where the release of monosaccharides from starch appears to play a key role in improving organoleptic characteristics. Additionally, the significant increase in aspartic and glutamic acid content observed after fermentation (6.78 to 7.35 and 10.08 to 11.50 g/100 g DW) may have contributed an umami taste dimension to FPF crepes, as glutamate liberation through proteolytic activity during fermentation is a well-documented driver of flavor improvement in fermented legume products [79]. Moreover, the observed decrease in lysine content may also have positively influenced taste perception, since this amino acid has been associated with bitter taste [55,79]. The improved acceptability of fermented flour could be also related to the reduction of the characteristic beany flavor. This off flavor is mainly associated with volatile aldehydes and ketones, especially hexanal, and 2-pentylfuran, generated through the oxidation of polyunsaturated fatty acids like linoleic acid [16,56,80]. Zipori et al. [80] found that fermentation of pea protein isolate with yeast cultures significantly reduced these compounds, decreasing hexanal levels to below its sensory odor threshold [16]. The reduction of these compounds may also explain the higher scores on taste attribute assigned by panelists to FPF compared to the UPF, although further studies on volatile fractions are required to confirm this mechanism. The improvement in consistency observed for FPF crepes relative to UPF (4.00 vs. 3.06) is consistent with the structural modifications undergone by the protein fraction during fermentation and heat treatment, which promoted protein denaturation and aggregation and increased water absorption capacity (127.73 to 136.30%), resulting in a more cohesive and structured texture upon cooking.

Notably, the starch-rich fraction crepes achieved the highest global acceptance scores regardless of fermentation (USF: 6.75; FSF: 6.31 vs. wheat control: 4.63), suggesting that the starch-rich fraction represents a particularly promising ingredient for direct application in cereal-based food products. In addition, fermentation has proved to be a useful strategy for the protein-rich fraction to reach comparable consumer acceptability, as reflected by the marked increase in overall score from unfermented to fermented protein-rich crepes (UPF: 4.00 vs. FPF: 5.94).

## 5. Study Limitations and Future Perspectives

Although the comparison between raw and fermented flour fractions allowed the changes associated with the fermentation process to be evaluated, the absence of a non-inoculated control subjected to the same incubation conditions limits the ability to distinguish the effects of *S. cerevisiae* from those potentially resulting from endogenous enzymatic activity or other non-fermentative processes. Future studies including non-inoculated controls would help to further elucidate the specific contribution of yeast fermentation to the observed changes.

The sensory evaluation was conducted with a relatively small panel of volunteers (n = 16), which represents a limitation of the study. In particular, attributes such as unpleasant odor and beany flavor may be subject to individual perception and may require a larger and more extensively trained panel for a more robust descriptive characterization. Future studies should therefore consider larger and formally trained sensory panels to further validate the sensory effects of fermentation.

## 6. Conclusions

In this study, it was hypothesized that the chemical, technological, and sensory properties of faba bean flours could be enhanced through solid-state fermentation (SSF) using baker’s yeast. To this end, *Saccharomyces cerevisiae* was tested on faba bean flours according to starch and protein contents. Under the current experimental conditions, the RFO content in the fermented flour was reduced to 22.5% (protein-rich fraction) and 39.2% (starch-rich fraction). The overall amino acid profile was largely preserved, indicating that nutritional quality was not compromised. Structural changes resulting from fermentation led to new interactions between protein residues, lipids and starch residues, thereby altering the techno-functional properties. The SSF increased water absorption capacity (6.7–12.4%) in both legume flours. In contrast, oil absorption capacity increased (22.7%) only in starch-rich flour but was not affected in the protein-rich flour. Therefore, these legume flours offer advantageous technological properties for industrial applications. It has also been demonstrated that SSF in protein-rich flour improved by 48.5% of its global acceptance. These findings highlight the potential of SSF as a viable strategy to broaden the applicability of faba bean flours in the development of plant-based alternatives. From a practical perspective, the use of baker’s yeast may facilitate the potential application of this approach due to the established use of *S. cerevisiae* in food fermentation processes. However, for industrial-scale implementation, further research is required to optimize fermentation process and to carefully control parameters as heat generation, O_2_/CO_2_ exchange, and moisture loss, which become increasingly difficult to manage at larger bed volumes and could compromise the reproducibility of the results obtained at laboratory scale. In addition, future studies should evaluate the behavior of these fermented flours in different food products to determine whether the improvements observed at the flour level translate into enhanced quality and functionality in the final products.

## Figures and Tables

**Table 1:** Ingredients used for five crepes formulations.

Ingredients	Wheat Based	USF Based	FSF Based	UPF Based	FPF Based
Wheat flour (g)	125	—	—	87.5	87.5
Faba bean flour (g)	—	125	125	37.5	37.5
Eggs (units)	1	1	1	1	1
Sugar (g)	10	10	10	10	10
Whole milk (mL)	250	250	250	250	250
Substitution rate (%)	—	100	100	30	30

USF: unfermented starch-rich faba bean flour; FSF: fermented starch-rich faba bean flour; UPF: unfermented protein-rich faba bean flour; FPF: fermented protein-rich faba bean flour.

**Table 2:** Physicochemical properties of unfermented and fermented starch- and protein-rich faba bean flours.

Parameter	USF	FSF	*Sign.*	UPF	FPF	*Sign.*
pH	6.41 ± 0.04	5.80 ± 0.02	*	6.37 ± 0.01	5.83 ± 0.02	*
Moisture (g/100 g flour)	5.70 ± 0.05	7.92 ± 0.09	*	7.73 ± 0.11	9.54 ± 0.10	*
Raw protein (g/100 g flour DW)	18.33 ± 0.31	19.06 ± 0.85	*	52.74 ± 0.81	51.22 ± 0.28	*
Raw fat (g/100 g flour DW)	1.71 ± 0.12	1.64 ± 0.16	ns	4.02 ± 0.61	4.97 ± 0.08	*
Raw carbohydrate (g/100 g flour DW)	77.51 ± 0.39	74.71 ± 0.89	*	37.29 ± 1.23	31.74 ± 0.32	*
Ash (g/100 g flour DW)	2.45 ± 0.05	2.61 ± 0.01	*	5.95 ± 0.02	5.62 ± 0.04	*

Values are given as mean ± standard deviation (n = 3). Comparisons between unfermented and fermented samples were performed by the Mann-Whitney test (*: *p* < 0.05; ns: not significant). USF: unfermented starch-rich faba bean flour; FSF: fermented starch-rich faba bean flour; UPF: unfermented protein-rich faba bean flour; FPF: fermented protein-rich faba bean flour.

**Table 3:** Digestible carbohydrates and organic acids in unfermented and fermented starch-rich and protein-rich faba bean flours.

Compound (g/100 g Flour DW)	USF	FSF	*Sign*.	UPF	FPF	*Sign*.
Starch	51.19 ± 1.28	47.34 ± 3.71	ns	15.67 ± 0.61	11.87 ± 0.21	*
Maltose	0.80 ± 0.16	0.63 ± 0.10	ns	0.88 ± 0.25	0.29 ± 0.06	*
Sucrose	2.87 ± 0.1	ND	*	2.44 ± 0.18	0.16 ± 0.09	*
Lactose	0.01 ± 0.01	0.02 ± 0.00	*	0.01 ± 0.01	0.01 ± 0.00	ns
Glucose	0.11 ± 0.02	0.55 ± 0.02	*	0.22 ± 0.04	1.18 ± 0.04	*
Fructose	ND	0.08 ± 0.02	*	0.01 ± 0.01	1.27 ± 0.05	*
Galactose	0.05 ± 0.00	0.59 ± 0.02	*	0.11 ± 0.01	0.31 ± 0.00	*
Total digestible carbohydrates	54.93 ± 1.13	49.21 ± 3.79	*	19.35 ± 0.51	15.01 ± 0.23	*
RFOs	1.48 ± 0.29	0.90 ± 0.04	*	1.51 ± 0.29	1.17 ± 0.08	ns
Malic acid	0.00 ± 0.00	0.09 ± 0.03	*	0.08 ± 0.02	0.12 ± 0.01	*
Citric acid	0.37 ± 0.05	0.28 ± 0.01	*	1.00 ± 0.06	0.98 ± 0.01	ns
L-lactic acid	0.01 ± 0.01	0.03 ± 0.01	*	0.02 ± 0.01	0.02 ± 0.00	ns
D-lactic acid †	0.00 ± 0.00	0.00 ± 0.00	ns	0.02 ± 0.01	0.00 ± 0.00	*
Acetic acid	0.00 ± 0.00	0.08 ± 0.01	*	0.05 ± 0.01	0.07 ± 0.01	ns
Total acids	0.38 ± 0.05	0.48 ± 0.03	*	1.15 ± 0.07	1.20 ± 0.02	ns

†: concentrations expressed in mg/100 g flour DW. Values are given as mean ± standard deviation (n = 3). ND: not detected. Comparisons between unfermented and fermented samples were performed by the Mann-Whitney test (*: *p* < 0.05; ns: not significant). USF: unfermented starch-rich faba bean flour; FSF: fermented starch-rich faba bean flour; UPF: unfermented protein-rich faba bean flour; FPF: fermented protein-rich faba bean flour.

**Table 4:** Amino acid composition of unfermented and fermented starch-rich and protein-rich faba bean flours.

Amino Acid (g/100 g Flour DW)	USF	FSF	*Sign*.	UPF	FPF	*Sign*.
Alanine	0.82 ± 0.11	1.03 ± 0.14	ns	2.42 ± 0.34	2.73 ± 0.39	ns
Valine	0.88 ± 0.12	1.11 ± 0.15	ns	2.71 ± 0.38	3.12 ± 0.43	ns
Isoleucine	0.80 ± 0.11	0.96 ± 0.13	ns	2.38 ± 0.34	2.81 ± 0.40	ns
Leucine	1.39 ± 0.19	1.74 ± 0.24	ns	4.54 ± 0.64	5.14 ± 0.72	ns
Phenylalanine	0.80 ± 0.11	0.99 ± 0.14	ns	2.63 ± 0.37	2.95 ± 0.41	ns
Methionine	0.00 ± 0.00	0.19 ± 0.03	*	0.42 ± 0.06	0.45 ± 0.06	ns
Tryptophan	0.19 ± 0.02	0.22 ± 0.02	ns	0.56 ± 0.06	0.60 ± 0.06	ns
Proline	0.67 ± 0.09	0.93 ± 0.13	ns	2.66 ± 0.37	2.83 ± 0.40	ns
Total Non-polar	5.55 ± 0.31	7.17 ± 0.39	*	18.32 ± 1.03	20.63 ± 1.16	ns
Glycine	0.82 ± 0.11	1.02 ± 0.14	ns	2.42 ± 0.34	2.74 ± 0.39	ns
Serine	0.92 ± 0.13	1.17 ± 0.16	ns	2.99 ± 0.42	3.32 ± 0.46	ns
Threonine	0.82 ± 0.12	0.88 ± 0.1	ns	2.07 ± 0.29	2.31 ± 0.32	ns
Cysteine + cystine	0.42 ± 0.06	0.24 ± 0.03	*	0.63 ± 0.09	0.55 ± 0.08	ns
Tyrosine	0.66 ± 0.09	0.65 ± 0.0	ns	2.01 ± 0.28	2.10 ± 0.30	ns
Total Polar (uncharged at pH 7)	3.64 ± 0.23	3.96 ± 0.2	ns	10.12 ± 0.68	11.02 ± 0.75	ns
Aspartic acid †	2.45 ± 0.02	2.55 ± 0.03	*	6.78 ± 0.03	7.35 ± 0.18	*
Glutamic acid †	2.93 ± 0.11	3.95 ± 0.05	*	10.08 ± 0.09	11.50 ± 0.07	*
Lysine	1.33 ± 0.01	1.49 ± 0.02	*	4.02 ± 0.05	3.68 ± 0.13	*
Arginine	1.83 ± 0.25	2.06 ± 0.29	ns	5.71 ± 0.80	6.26 ± 0.87	ns
Histidine	0.59 ± 0.08	0.59 ± 0.08	ns	1.51 ± 0.21	1.73 ± 0.24	ns
Total Polar (charged at pH 7)	9.13 ± 0.29	10.64 ± 0.30	*	28.10 ± 0.83	30.52 ± 0.93	*

†: concentrations refer to the sum of Aspartic Acid + Asparagine and Glutamic Acid + Glutamine Values are given as mean ± standard deviation (n = 3). Comparisons between unfermented and fermented samples were performed by the Mann-Whitney test (*: *p* < 0.05; ns: not significant). USF: unfermented starch-rich faba bean flour; FSF: fermented starch-rich faba bean flour; UPF: unfermented protein-rich faba bean flour; FPF: fermented protein-rich faba bean flour.

**Table 5:** Techno-functional properties of unfermented and fermented starch-rich and protein-rich faba bean flours.

Property (%)	USF	FSF	*Sign*.	UPF	FPF	*Sign*.
WAC	160.84 ± 5.48	180.80 ± 2.39	*	127.73 ± 1.85	136.30 ± 0.85	*
WS	21.88 ± 0.13	12.30 ± 0.59	*	54.79 ± 0.25	41.77 ± 0.33	*
OAC	93.59 ± 0.56	114.77 ± 2.74	*	121.84 ± 1.83	117.57 ± 4.80	ns
EA	57.50 ± 0.00	51.25 ± 0.00	*	54.96 ± 1.63	56.25 ± 1.25	ns
ES	54.17 ± 0.72	50.42 ± 0.72	*	51.03 ± 2.20	55.00 ± 1.25	*
FC	88.89 ± 3.85	84.45 ± 3.85	ns	164.44 ± 3.85	133.33 ± 6.67	*
FS	74.10 ± 2.35	84.39 ± 3.89	*	68.68 ± 4.40	74.09 ± 2.82	ns

Values are given as mean ± standard deviation (n = 3). Comparisons between unfermented and fermented samples were performed by the Mann-Whitney test (*: *p* < 0.05; ns: not significant). USF: unfermented starch-rich faba bean flour; FSF: fermented starch-rich faba bean flour; UPF: unfermented protein-rich faba bean flour; FPF: fermented protein-rich faba bean flour.

**Table 6:** Least gelling concentration of unfermented and fermented starch-rich and protein-rich faba bean flours.

Concentration (% *w*/*v*)	USF	FSF	UPF	FPF
10	1	1	2	1
12	1	1	3	3
14	1	1	3	3
16	1	1	3	3
18	1	2	3	3
20	1	3	3	3

(1) liquid, no gel formation, (2) weak gel, the solution flowed with some resistance, (3) gel formation, when the solution did not flow when the tube was inverted. USF: unfermented starch-rich faba bean flour; FSF: fermented starch-rich faba bean flour; UPF: unfermented protein-rich faba bean flour; FPF: fermented protein-rich faba bean flour.

## Data Availability

The original contributions presented in this study are included in the article/Supplementary Materials. Further inquiries can be directed to the corresponding author.

## Supplementary Materials

The following supporting information can be downloaded at: https://www.mdpi.com/article/10.3390/foods15173110/s1, Figure S1: Appearance of faba bean flours: (a) high in starch (left: unfermented; right: fermented); (b) high in protein (left: unfermented; right: fermented); Figure S2: Appearance of crepes prepared with (a) wheat flour; (b) flour from fermented starch-rich faba bean; (c) flour from fermented protein-rich faba bean; Table S1: Sensory quality attributes of crepes formulated with wheat flour (control) and starch-rich faba bean flours; Table S2: Sensory quality attributes of crepes formulated with wheat flour (control) and protein-rich faba bean flours.

## Abbreviations

The following abbreviations are used in this manuscript:

SF	Starch-rich faba bean flour
PF	Protein-rich faba bean flour
USF	Unfermented starch-rich faba bean flour
FSF	Fermented starch-rich faba bean flour
UPF	Unfermented protein-rich faba bean flour
FPF	Fermented protein-rich faba bean flour
SSF	Solid-state fermentation
RFOs	Raffinose Family Oligosaccharides
WAC	Water Absorption Capacity
WS	Water Solubility
OAC	Oil absorption capacity
EA	Emulsifying Activity
ES	Emulsion Stability
FC	Foaming Capacity
FS	Foam Stability
LGC	Least Gelation Concentration
GMO	Genetically Modified Organisms
DW	Dry Weight
