# Supplementary material for: Solid-State Fermentation with Saccharomyces cerevisae: A Strategy to Modulate the Properties of Faba Bean Flours
Source: Foods. 2026 Sep 1;15(17):3110. doi: 10.3390/foods15173110 (PMC13565044; doi:10.3390/foods15173110)
Supplement: Supplementary file 1 [file foods-15-03110-s001.zip › foods-4495090-supplementary.pdf]

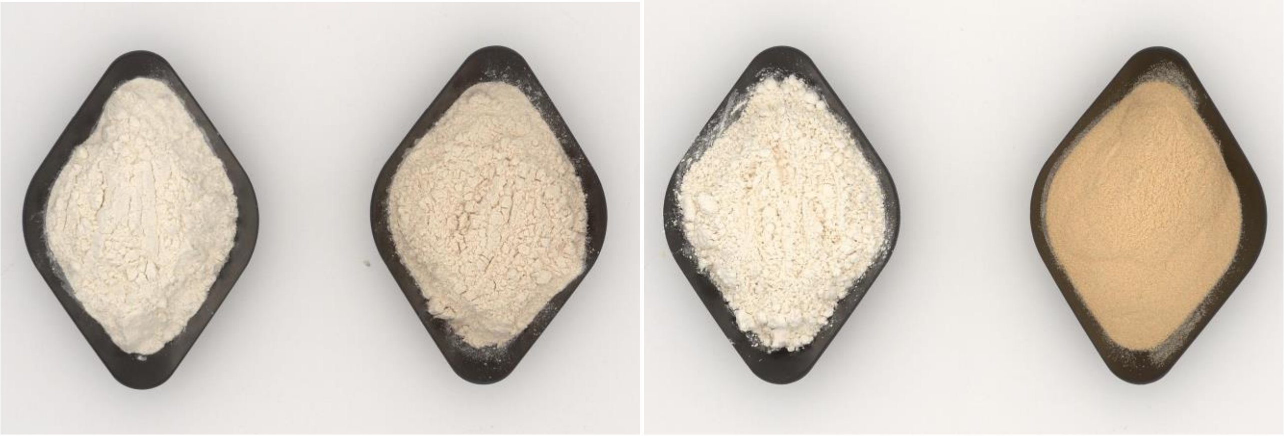

(a) **(b)**

Figure S1. Appearance of faba bean flours: (a) high in starch (left: unfermented; right: fermented); (b) high in protein (left: unfermented; right: fermented)

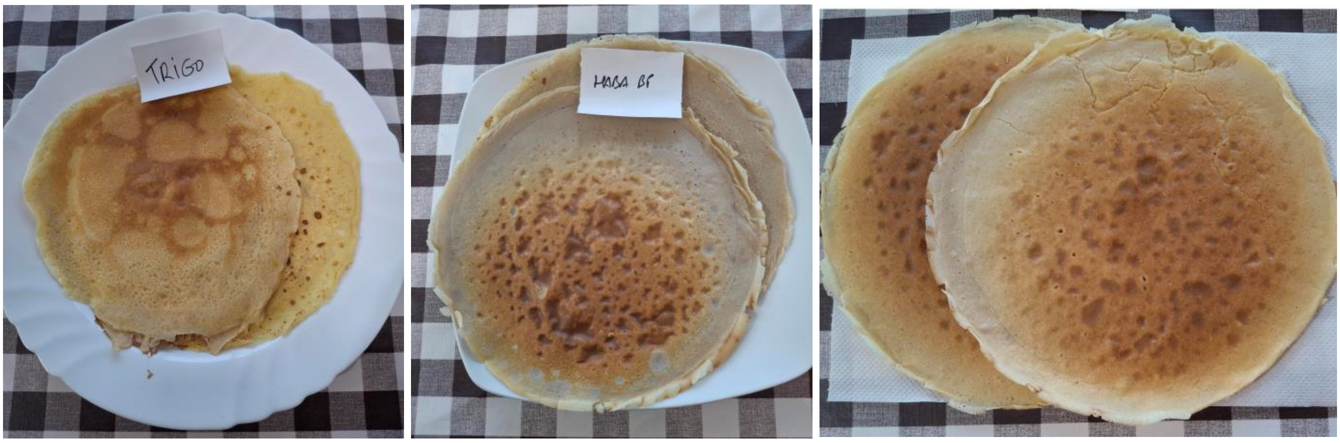

(a) **(b)** **(c)**

Figure S2. Appearance of crepes prepared with (a) wheat flour; (b) flour from fermented starch-rich faba bean; (c) flour from fermented protein-rich faba bean.

Table S1. Sensory quality attributes of crepes formulated with wheat flour (control) and starch-rich faba bean flours.

Attribute	Wheat flour	Unfermented starch-rich flour	Fermented starch-rich flour
Appearance	3.31 ± 0.70^{a}	4.06 ± 0.77^{bA}	3.56 ± 1.15^{aA}
Odor intensity	2.50 ± 1.15^{a}	2.44 ± 0.96^{a}	2.69 ± 0.70^{a}
Unpleasant Odor	1.69 ± 0.79^{a}	1.19 ± 0.40a^{A}	1.81 ± 0.98^{aB}
Taste	2.81 ± 1.17^{a}	4.06 ± 0.68^{bA}	3.75 ± 0.77^{bA}
Consistency	2.38 ± 1.02^{a}	4.00 ± 0.82^{bA}	3.75 ± 0.77^{bA}
Stickiness	1.69 ± 0.79^{a}	1.81 ± 0.91^{a}	1.63 ± 0.72^{a}
Global acceptance	4.63 ± 1.36^{a}	6.75 ± 1.34^{bA}	6.31 ± 1.25^{bA}

Values are given as mean ± standard deviation (n = 16). Different lowercase in the same row indicates statistically significant differences compared to the control sample (wheat flour) and uppercase indicates statistically significant differences between unfermented and fermented flours according to the Mann-Whitney test ($p < 0.05$).

Table S2. Sensory quality attributes of crepes formulated with wheat flour (control) and protein-rich faba bean flours.

Attribute	Wheat flour	Unfermented protein-rich flour	Fermented protein-rich flour
Appearance	3.31 ± 0.70^{a}	3.25 ± 1.24^{a}	4.00 ± 0.73^{a}
Odor intensity	2.50 ± 1.15^{a}	3.56 ± 0.51^{bA}	3.00 ± 1.03^{bB}
Unpleasant Odor	1.69 ± 0.79^{a}	2.81 ± 0.91^{b}	2.19 ± 1.11^{b}
Taste	2.81 ± 1.17^{a}	2.13 ± 0.89^{bA}	3.44 ± 0.81^{bB}
Consistency	2.38 ± 1.02^{a}	3.06 ± 0.85^{bA}	4.00 ± 0.63^{bB}
Stickiness	1.69 ± 0.79^{a}	2.06 ± 0.93^{a}	2.06 ± 0.85^{a}
Global acceptance	4.63 ± 1.36^{a}	4.00 ± 1.10^{aA}	5.94 ± 1.53^{bB}

Values are given as mean ± standard deviation (n = 16). Different lowercase in the same row indicates statistically significant differences compared to the control sample (wheat flour) and uppercase indicates statistically significant differences between unfermented and fermented flours according to the Mann-Whitney test ($p < 0.05$).
